# Introducing the Brassica Information Portal: Towards integrating genotypic and phenotypic Brassica crop data

**DOI:** 10.12688/f1000research.11301.2

**Published:** 2017-11-15

**Authors:** Annemarie H. Eckes, Tomasz Gubała, Piotr Nowakowski, Tomasz Szymczyszyn, Rachel Wells, Judith A. Irwin, Carlos Horro, John M. Hancock, Graham King, Sarah C. Dyer, Wiktor Jurkowski

**Affiliations:** 1 Earlham Institute, Norwich, NR4 7UZ, UK; 2 Academic Computer Centre CYFRONET, AGH University of Science and Technology, Kraków, 30-059, Poland; 3 John Innes Centre, Norwich, NR4 7UH, UK; 4 Southern Cross Plant Science, Southern Cross University, Lismore, NSW, 2480, Australia; 5 NIAB, Cambridge, CB3 0LE, UK

**Keywords:** Brassica, phenotypic trait data, data integration, FAIR data, phenotyping API, crop ontology, genotype-phenotype association, enhanced breeding

## Abstract

The Brassica Information Portal (BIP) is a centralised repository for brassica phenotypic data. The site hosts trait data associated with brassica research and breeding experiments conducted on brassica crops, that are used as oilseeds, vegetables, livestock forage and fodder and for biofuels. A key feature is the explicit management of meta-data describing the provenance and relationships between experimental plant materials, as well as trial design and trait descriptors. BIP is an open access and open source project, built on the schema of CropStoreDB, and as such can provide trait data management strategies for any crop data. A new user interface and programmatic submission/retrieval system helps to simplify data access for researchers, breeders and other end-users. BIP opens up the opportunity to apply integrative, cross-project analyses to data generated by the Brassica Research Community. Here, we present a short description of the current status of the repository.

## Introduction

*Brassica L.* is a diverse genus that includes six species cultivated as important crops, which yield edible oil and condiment seeds, roots, leaves, stems, vegetative and floral buds and meristems ^1^. Due to their wide adaptation and ability to thrive under varying agroclimatic conditions ^2^, brassica crops are grown throughout the world for food, animal forage and fodder and industrial applications.

Given the importance of *Brassica* species worldwide, integrated approaches to research and plant breeding for crop improvement are required to address future global challenges, such as satisfying increased demand for higher quality and nutritious food, the reduction of waste, and producing predictable yields in a more variable environment with sustainable, lower input cultivation systems. Researchers have quantified many aspects of rassica phenotypic diversity to assist in crop improvement, including yield in response to fertiliser input, degree of resilience to pathogens and pests and the composition of industrially important compounds. The ability to host the accumulation of diverse data generated from such experiments in an open, persistent and accessible database is expected to promote the downstream comparison of trait measurements. In particular, there is scope to calculate genotype-phenotype (G X E) associations across multiple studies in meta-analyses, as well as identifying previously unrecorded correlations. In combination with the massive increase in availability of genomic data, this is likely to help unravel underlying molecular mechanisms and pleiotropic or epistatic interactions. Trait resolution in the context of marker associations is particularly important for plant breeders and researchers involved in pre-breeding, which can accelerate the generation of new crop varieties through use of genome-wide association studies (GWAS). For example, meta-analysis of trait data from multiple studies across different populations or environments can help confirm the location of key areas of the genome, or assist and help manage potential trait trade-offs between beneficial and deleterious alleles due to linkage drag. The ability to associate phenotypic data with specific genomic regions, transcribed sequences regulated in *cis* or *trans* provides the potential to expedite the integration of favourable alleles via marker-assisted or genomic selection. However, few cohesive plant phenotype databases exist, despite the importance of phenotypic data to render meaning to genomic sequence data.

Maintenance of easily accessible and reusable plant phenotypic data is challenging because, in contrast to standardised approaches used in sequence analysis, small variations in the protocols used to assess traits can lead to significant quantitative and sometime qualitative variation in values recorded. Moreover, beyond more recent phenomic approaches, the trait descriptors, protocols and methods for most plant phenotypes are seldom captured automatically, and often there is no consensus on methodologies to record any given trait. In addition, there is a lack of controlled vocabularies to describe phenotyping metadata adequately. One possible reason is that, compared to publishing sequencing data in databases widely used by the research community, such as GeneBank ^3^, ArrayExpress ^4^, GEO (Gene Expression Omnibus) ^5^, ENA (European Nucleotide Archive) ^6^ and SRA (Sequence Read Archive) ^7^, there is no requirement to submit phenotyping data to a public repository prior to peer-reviewed publications. There is, to date, no single plant phenotypic database recommended by Nature’s Scientific Data. Compared to sequence data, the formal description of trait and associated meta-data tends to be more complex and, in the absence of robust generic ontologies, crop or species specific.

Data re-use and re-analysis can lead to new knowledge insights and understanding. For example, readily available datasets retrieved from a database can serve to explore existing knowledge and identify gaps, as a starting point for initial hypothesis testing, and to help with selecting new research directions. Additionally, databases can serve as tools for collaboration across countries, fields or areas of expertise and strengthening links between public sector research and private industry.

Existing brassica-related databases and analysis tools such as BRAD ^8^ mostly focus on brassica genomic information. Other databases are project- or institute-specific, such as PlabiDB ^9^, a crop genomic platform that, among others, hosts genomic data from *Brassica napus*, and GnpIS ^10^ that, besides genomic information, hosts some phenotype data that are not publicly available. While these databases are useful in their own context, they do not focus on establishing or demonstrate the value of community-wide phenotypic data standards, and therefore do not encourage universal trait sharing and data re-use.

Resources, such as TAIR (arabidopsis.org) ^11^, Araport (araport.org) ^12^ and the 1001 Genomes Project ^13^, provide plant genomic data and analysis tools for the arabidopsis research community. Due to the close evolutionary and taxonomic relatedness of the model plant *Arabidopsis thaliana* in the *Brassicaceae*, arabidopsis-related databases or platforms for genotype and phenotype information provide valuable insights into the underlying *Brassica* biology, and potential insights to guide crop analysis tools and resources of the future. However, current Arabidopsis resources are not directly applicable to recording genotype-phenotype specific traits for brassica crops.

CropStoreDB (cropstoredb.org) ^14, 15^, was initially developed to provide a pre-processing curation framework to assist in collation and dissemination of datasets from crop-specific research communities. Its initial use case was for brassica phenotypic and genetic map data, including sequence-tagged markers associated with a reference integrated linkage map for *B. napus* ^16^, and data from Integrated Marker System for Oilseed Rape Breeding ^17^ and and BrassicaDB ^18, 19^ projects, along with a range of mineral analyses from *B. oleracea* ^20– 22^. CropStoreDB was a central component of InterStoreDB ^14^, which demonstrated the means by which navigating between a set of interlinked databases could assist in associating phenotypic traits and quantitative trait loci (QTL) to causative regions of the underlying genome.

The Brassica Information Portal builds on the CropStoreDB schema, and provides the technologies to enable efficient storage, classification and management of phenotyping data according to FAIR principles ^23^. Its unique features include support for use of controlled vocabularies and ontologies, cross-references to sequence information and other raw data, user-friendly wizard-based submissions, efficient search and Application Programming Interface (API) based data submission and retrieval. In addition, submitted data can be kept private for a user-defined period of time. Visual data exploration and tools for associative analysis are intended in the future releases, to provide a valuable set of features for the brassica research community.

## Methods

To address the challenges facing a phenotypic trait database, the BIP has implemented rigorous use of ontologies and standards. For that purpose we created a dictionary of brassica-specific traits created according to CropOntology ^24^. The CropOntology phenotype annotation model is based on the combination of “trait method and scale”, as it is grounded in formats of datasets used by breeder communities ^24^. This makes it especially suitable for describing the properties and relationships between traits that are typically assessed in pre-breeding plant material, and likely to be hosted in the portal. The level of detail incorporated in the data fields used to describe and define traits makes it possible for end users to select meaningful traits for downstream comparative analysis. Since, the CropOntology encourages a way of defining a trait to facilitate meta-analysis, integrated data analysis and data discovery, it aligns well with the main aims of BIP beyond a data storage repository.

Further incorporation of ontology terms includes those for plant anatomy derived from Plant Ontology ^25^ and taxonomical classification from Gramene’s Taxonomy Ontology ^26^. Registered cultivar names are curated, and submissions are checked against this curated list.

Initial minimal requirements for trial, population and trait description have been identified, and are made mandatory during dataset submission, thus ensuring meaningful experimental meta-data are associated with the trait scores. BIP development is benefiting from discussions on standard minimal requirements for plant phenotyping experiment (MIAPPE) data, which are currently ongoing as part of the Excelerate project run by the ELIXIR consortium. MIAPPE functions as a check-list of attributes that can be used to describe each experimental unit ^27, 28^ and allows the representation of the most useful information captured by researchers to allow comprehensive data sharing and reuse. A similar approach has been applied previously for other data types, including gene expression microarrays (MIAME), sequencing (GSC), metabolomics (MSI), and proteomics (MIAPE).

In BIP, we are focusing on information related to recording Project, Trial, Sample, Location, Treatment and Trial Environment and associated design factors. Our contribution includes the definition of MIAPPE fields and proposal of ontologies to ensure there is standardised content to describe brassica phenotyping experiments mapped to these requirements. With regard to file types, we have adopted standard input and output files in the .csv format, as it is readable by most software tools and humans alike, and is likely to persist. Further, the API allows for machine readable .json output format.

The content of the database includes experimental population data with associated member plant lines and diversity sets with plant lines representing cultivars or other *ex-situ* genetic resource material. Linked to this hierarchical relationship of plant accessions and scoring units are trait scores with associated experimental and organisational metadata. As this repository has inherited its structure and content from CropStoreDB, it also hosts historical QTL and linkage map data linked to experimental segregating populations and trait data. For obvious reasons we do not intend BIP to incorporate sequencing data managed elsewhere. However, BIP continues to host genotyping data generated through modern DNA, RNA sequencing or genotyping arrays, with reference between SNP and other markers to unique sequence identifiers in other repositories. The BIP currently hosts data from numerous pre-breeding programmes that include 22 plant trials, with 139,000 trait scores from 188 different cultivars and 26,100 plant lines.

While the current version of the BIP repository lays the foundation for more extensive integration of genotype and phenotype information and tools, it is already integrated with TGAC Browser ^29^ to allow cross-linking of single nucleotide polymorphisms (SNPs) of interest to lines/cultivars in the BIP that may carry the corresponding SNP.

### Implementation

There are three main building blocks that constitute the BIP. The first element is the relational database, which stores and manages all BIP data. It is implemented using PostgreSQL RDBMS technology. BIP’s initial datasets and the database schema were obtained from the CropStoreDB (schema v9.01) system and then underwent several transformations, including translation from the original MySQL format to the target PostgreSQL format, i.e. introduction of formal foreign keys and presence constraints, and a series of data curation procedures to ensure referential integrity. The BIP database is the core of the system, enacting all data management operations, issued both through the web interface and the REST API.

**Figure 1.**
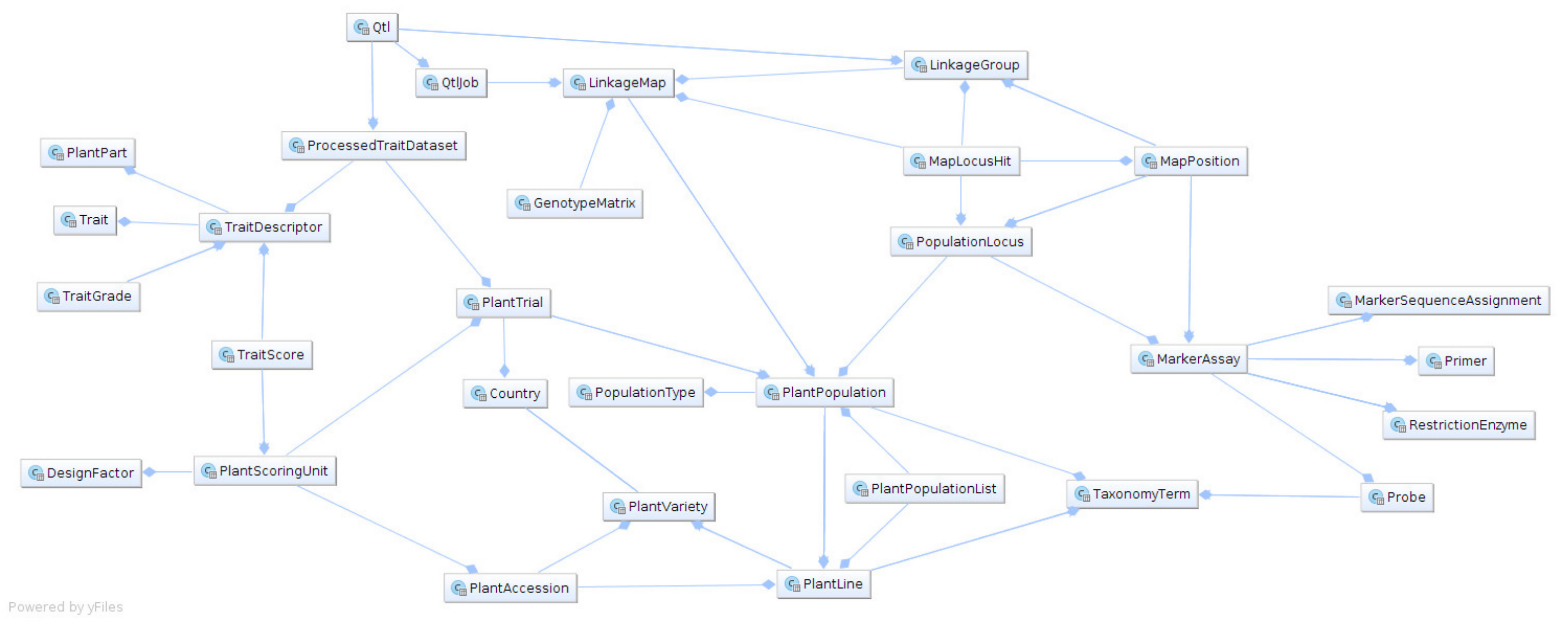
BIP database model. The diagram provides an overview on all tables in the BIP database, and the relations between them. Some “operational” tables, which are used only internally by the system (e.g. for access rights), were omitted.

The decision to transfer data to PostgreSQL was taken as it delivers more advanced, modern capabilities of data management than MySQL, while still being an open source and a free-of-charge solution. The overview of the database schema is presented in Figure 1.

**Figure 2.**
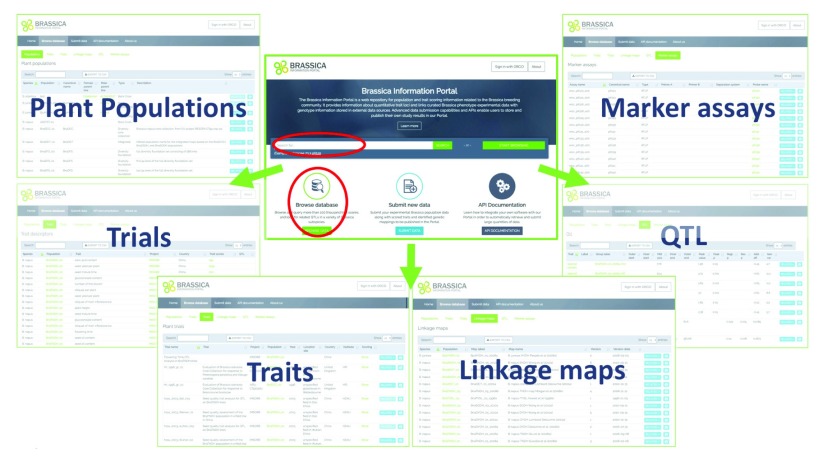
Access to the database via the web interface is possible either by global search across all indexed database fields (upper red circle), or through targeting specific content/tables by following the link on “Browse database” (lower red circle).

The second element is built on top of the database and is a modern web application. Its purpose is threefold: (i) it delivers advanced data browsing interfaces, which allow for data ordering, filtering and export to CSV documents available to all users without registration; see Figure 2 (ii) it implements data submission wizards (for registered users only), explained in detail in the section below (iii) and it gives the possibility of managing data through the API, which supports reading, loading, querying, searching, creating and publishing data.

The web application was created using the Ruby on Rails framework. All web requests are channelled through the Nginx web server, which in turn routes them to a web application logic server (a so-called reverse proxy setup). All traffic is secured with an encrypted HTTPS protocol. User registration is implemented using the third party authentication solution ( ORCiD).

The third constituent element of the BIP is the data indexing and full-text search engine. It analyses all records in every table of the database, including all new data being submitted by users, and creates a search index. The BIP web search functionality (present on the front page of the website; see Figure 2) and its API search capability (available via programmatic access to registered users) is integrated with the search engine in order to deliver the arbitrary term search. For every submitted search term, the engine first looks the term up in the index, and if found, responds with a set of data-table records that include this term. This element of the BIP is created using Elasticsearch technology.

**Figure 3.**
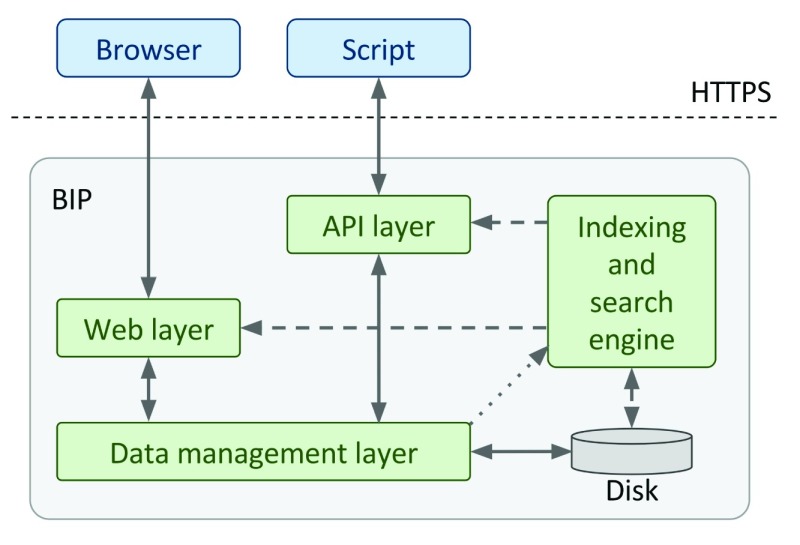
BIP architecture diagram. Users initiate actions with either their web browsers or API clients (blue elements). All requests go through respective access control layers (these ensure e.g. that a given set of data is accessible to the user), before they contact the data management layer to retrieve (or commit) data from (to) the database. Access mode is read/write (solid arrows). Additionally the search engine could be required to search through gathered data (dashed arrows) - it’s a read-only access mode. Finally, the data layer informs the search engine about any new data to be indexed (the dotted arrow).

All three elements are presented in Figure 3, together with respective data transfer channels between them. In terms of deployment, the database system, the web application logic server, and the indexing and search engine all reside within a single system. If, in the future, the scale of the data managed by the BIP becomes too high for current machine capabilities, it is possible to decouple these elements in order to distribute the load on several physical servers.

### Operation

BIP operates at bip.earlham.ac.uk/ and is publicly accessible at this address, as a web resource. However, it is possible to use the BIP open source to deploy one’s own BIP instance. In such a case, BIP will require a server, either physical or virtual, with at least two contemporary CPU cores, and at least four gigabytes of operational memory. Due to its internal architecture, described in the previous section, two processor cores are required to achieve a smooth user experience along with simultaneous maintenance of fast database and indexing engine responses. Since the DB and the web application itself makes a heavy use of disk caching mechanisms to provide faster response to user queries, it is also beneficial, though not absolutely required, to equip the server with fast hard drives. The storage space needed depends on the amount of data one plans to deposit in the instance of BIP, but the application itself has no high requirements in this respect, and 6 GB of storage space should be sufficient for both the operating system and all the components of BIP. Any up-to-date Linux operating system will be adequate to host BIP.

## Use of the BIP

### Content navigation

Several tables host interlinked population, trial, trait score, linkage map, QTL and marker assay data, and are accessible via the web-interface, either by performing a global search (across all indexed database fields), or by browsing the database for specific tables ( Figure 2).

**Figure 4.**
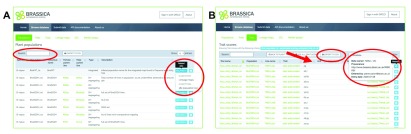
Multiple mechanisms to find, navigate and access data in BIP. **A**) refined querying and access to related data **B**) reordering of results, function for bulk download of data with accompanying metadata, display of the meta-data.

Accessing related data is facilitated by following the “Related” button in case of the Populations table, or by clicking onto a cross-linked field within the table (these appear green; Figure 4A). On top of each table, a search bar can query for specific components within the table ( Figure 4A). Organisational and provenance metadata are made visible by clicking the blue information button for each row ( Figure 4B). Each column can be sorted, resulting in global reordering across the whole table ( Figure 4B, red arrow). The visible content of the tables can be downloaded as a .csv file, by clicking on the respective button on top of the table ( Figure 4B).

### Submission and download of data

Experimental data are generally recorded in spreadsheet format. Therefore, both the web form wizard provided within the resource, and the available Ruby script client, use spreadsheet formats as templates to assist the user in uploading the data. Data are submitted in two separate logical batches. First, information about the experimental plant population needs to be submitted. Second, trait data (variates) incorporating design factors from Trials performed on these plants are submitted during Trait Scoring Submission. This enables the submission of multiple trials per experimental Plant Population.

**Figure 5.**
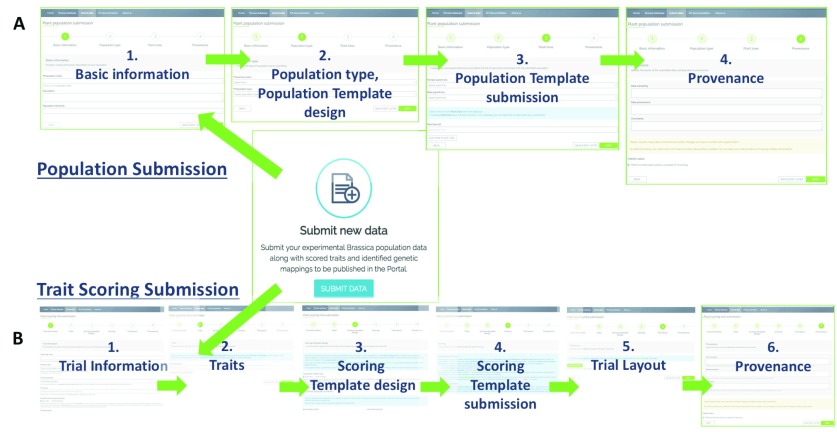
Wizards for Population Submission **A**) and Trait Scoring Submission **B**) comprise number of steps collecting required meta-data, to generate customised templates for data collection and to finally submit complete data sets.

A wizard-based submission of Population and Trial Data data guides the user through all compulsory and optional field contents that can be submitted to the database. During Population Submission ( Figure 5A), a four-step process asks the user to enter population related information, including the submission of the actual lines and associated metadata in a .csv template designed in step 3. The purpose of the template is to make it easier to appropriately format large data sets and avoids spelling mistakes in the uploaded file’s header. The standard template used during Population Submission is shown in Supplementary Figure 1B. Plant variety names are checked for their existence and correct spelling against the database. The final step is the submission of data provenance information. Compulsory metadata for Population Submissions is listed in Supplementary Table 1.

During Trial Submission ( Figure 5B), the first steps involve entering trial related information, before the traits studied in the trial are selected or defined in step 2. To avoid duplicated traits, the interface suggests traits based on an elastic search using two or more letters typed by the user. If traits are unavailable, the user can define and add them manually (the Plant and Trait Ontology vocabulary is provided to assist the user in this task). A high quality of trait definition and trait measurement-interoperability between experiments is ensured by interposing a human curator between submitter and database.

For the submission of trait measurements, the user designs a .csv template in step 3 (see Supplementary Figure 1A for an example template), and then submits the completed .csv file in step 4. Depending on the type of data submitted (raw vs. analysed data), the template can differ in the fields to be filled out. Compulsory metadata for Trial Submissions is listed in Supplementary Table 1. Finally, the user can upload an image presenting the experimental layout (for instance, the greenhouse plant pot setup on benches) in the case of raw data submission, and fill out provenance metadata in step 6. Whether data are submitted using the wizard or the API, the same metadata is compulsory.

Programmatic submission is executed using POST requests through the BIP API. The query exposes the part of the database to which the user wishes to submit data. Content to be submitted needs to be assigned a corresponding fields in the database and is presented as files in .json format. Programmatic extraction is also API based. GET requests placed to the database can be more comprehensive or specific than the interface-based strategy. These are then output in .json format, ready to feed into analytic workflows run in, for example, Python. Full API documentation is available on the portal's website, including all table fields beyond those that are compulsory.

A Ruby submission client facilitates the automated submission of new experimental populations and trial data. It parses an input .csv file searching for the elements (input columns) to be submitted. These input columns need previously to have been mapped against the database fields by the registered user. For each line in the .csv, new entries are created in the database and this content is then submitted to the database using the BIP API. Using the Ruby client tool is not required - it is provided only as a convenient alternative to users who prefer scripting approach to data submission and analysis.

The registered user is entitled to upload data directly to the database, which may then become public within seconds of submission. However, to ensure confidentiality of data for an ongoing project, at user’s discretion, a submission embargo can keep data private for a defined period. Once public and older than the revocation period, datasets could be assigned (on user’s demand) with individual DOIs, which act as a reference in publications.

Data download can be performed via the web interface and the API. Using the interface, the user can download any visible table, by clicking the "Export to CSV" button on top of the tables. This downloads the complete table in .csv format. Via the API, the user can use GET requests to interact with the database tables directly. This sometimes enables the user to retrieve more information than is displayed at the interface. The queries can be crafted according to the user’s needs for data. Example Ruby clients are available on Github for modification and retrieval of more complex data.

## Conclusions

The Brassica Information Portal (BIP) has been designed as an open-access, open-source, phenotype data entry, storage and retrieval resource and will facilitate addition of other features in the future. The general motivation for the BIP was to develop technologies to maximise efficient use of accumulated phenotyping data. The creation of the database and its standards lays the foundation for brassica genotype-phenotype studies, where phenotypic data may be sourced from this reference repository.

Availability of a repository for integrative phenotype data should encourage the brassica research community to store trait data in an open access repository, in a similar manner as for other data types, such as sequence, metabolomic and protein structure data, and share it with other members of the wider research, development and end-use community. Ultimately, the Portal will facilitate understanding of *Brassica* diversity and trait variation for future research, breeding programmes and crop improvement.

In many areas BIP complies with the FAIR guiding principles ^23^. For example, to enhance interoperability and re-usability of the data we adhere to controlled trait and taxonomy vocabularies, and by requiring rich meta-data in the data submission process. Furthermore, to enhance interoperability and accessibility, the BIP API is using .json for data download to increase both automatic integration into analysis tools, external services or APIs e.g. BrAPI (Breeding Application Programming Interface) as well as to ensure a human-readable data format. Thus far, we have made it possible to cross-link plant Accession data with associated Sequence data at SRA. Finally, as and additional step to enhance accessibility, we are using the common OAuth solution with ORCiD relevant for individual researchers and organisations. More work is needed to establish full FAIRness of brassica phenotyping data, with some features still under development and due to be introduced in the future releases of BIP.

With instant submission to the repository, it remains the primary responsibility of the submitter to guarantee the quality of the data. However, cross-linking with existing ontologies and spelling control holds errors in check prior to submission. Further, completion of mandatory data fields during submission ensure metadata meet minimal requirements for meaningful data interpretation in the future. Furthermore, each submission is granted a one week revocation period. During that time, the submitting author is asked to perform all important data quality checks, and in case of any discrepancies or inconsistencies are found, the author is able to revoke the publication, repair the submitted dataset, and publish it again. After a week has elapsed since the submission, the data are made read-only, and it can no longer be altered or removed. Moreover, it can then be accessed and quoted by other users as soon as the embargo is lifted. The author may request a DOI number assigned to the submission, so it is possible to cite it in publications.

Future plans include supporting the submission of imaging data to CyVerse, allowing BIP to serve as a single entry-point for multiple data types relevant to crop improvement studies. At the same time, visualisation tools will be developed to call and display the CyVerse-hosted images from within the BIP interface. This will include 1) images of single plant parts underlying definitions of specific traits; 2) time lapse images of single plants; and 3) time lapse images of field trials generated by high-throughput techniques. Statistical analysis and navigation of phenotypic data will be possible with incorporation of R/Shiny applications run directly on the BIP server. For example, association studies could be performed with uploaded genotyping data matching selected plant lines with corresponding trait measurements available in the BIP. More computationally costly operations will be supported through development and incorporation of analytical workflows executed externally, e.g. using CyVerse infrastructure. Additionally, linkage markers recorded in the database will be cross-linked with their corresponding sequences within EnsemblPlants ^30^. This will make it possible to visualise the location of the markers in context with the *Brassica* genome sequence and genes in close proximity.

Finally, the BIP API will be further refined by implementing additional content checks, e.g. by cross-linking to Global and European cultivar registers: Plant variety database, Community Plant Variety Office,CPVO Organisation for Economic Co-operation and Development (OECD) Variety List Query and to comply with ELIXIR standards (currently under development) to facilitate common data exchange and communication with other bioinformatics resources.

## Summary

We have introduced the Brassica Information Portal, a web repository for *Brassica* phenotype data. This article describes the current state of development and future plans. We describe the back end database schema and the front end web-interface. Also, we describe programmatic and wizard-based submission of Population and Trial data together with fields compulsory for submission to the repository. It was originally intended that this repository meet the needs of the Brassica Research Community in the UK for relevant experimental data. However, it is apparent from discussions within the Multinational Brassica Genome Project that it can now fulfil far wider community infrastructure requirements. We therefore hope that BIP encourages data sharing and re-use by helping to integrate and harmonise datasets generated worldwide.

## Software availability

Brassica Information Portal can be accessed from: bip.earlham.ac.uk

Latest source code: https://github.com/TGAC/brassica

Archived source code as at time of publication: https://doi.org/10.5281/zenodo.466050

License: GNU 3.0

## Supplementary material

**Supplementary Figure 1: Examples of .csv templates generated during (A) Trait Scoring Submission and (B) Population Submission.** The latter can change substantially depending on the type of data submitted. In this case raw data is being submitted as the template asks for information on the trial design.

**Supplementary Table 1: List of fields that are compulsory during the submission of Populations and Trial (Trait Scoring) data.**
