# Supplementary figures and images for: Introducing the Brassica Information Portal: Towards integrating genotypic and phenotypic Brassica crop data
Source: F1000Res. 2017 Nov 15;6:465. Originally published 2017 Apr 12. [Version 2] doi: 10.12688/f1000research.11301.2 (PMC5428495; doi:10.12688/f1000research.11301.2)

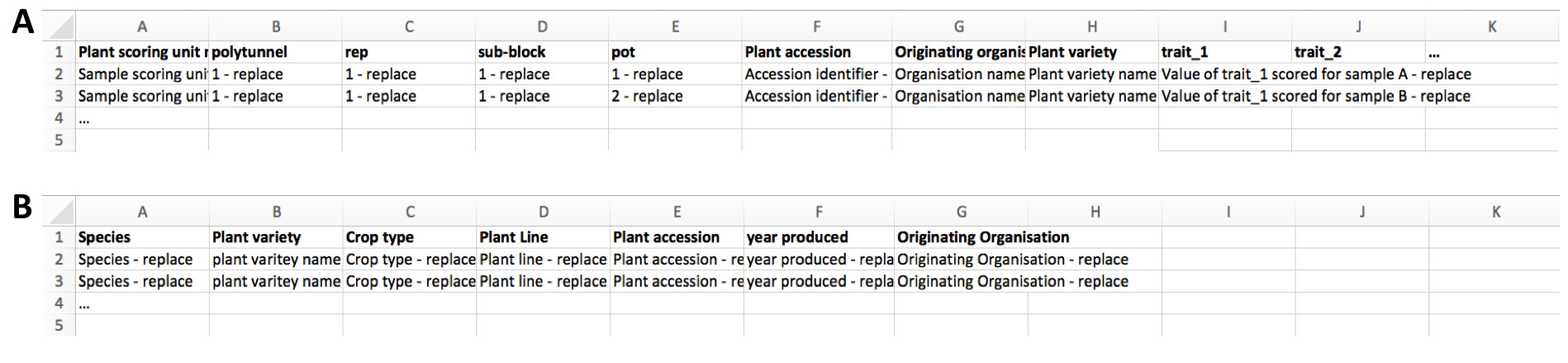

Supplement: Supplementary file 1 [file f1000research-6-14007-s0000.tgz › f149e09a-6c28-4f5a-b62c-4b4b9d38b79b.jpg]
