# Supplementary material for: Introducing the Brassica Information Portal: Towards integrating genotypic and phenotypic Brassica crop data
Source: F1000Res. 2017 Nov 15;6:465. Originally published 2017 Apr 12. [Version 2] doi: 10.12688/f1000research.11301.2 (PMC5428495; doi:10.12688/f1000research.11301.2)
Supplement: Supplementary file 2 [file f1000research-6-14007-s0001.tgz › efbd935b-1494-48e5-a3ee-062529da2c36.docx]

| Population Submission | Trait Scoring Submission |
| --- | --- |
| 1. Population Information | 1. **Trial Information** |
| Plant Population Name | Plant trial name |
| Description | Project name |
|  | Plant Population Name |
|  | Trial year |
|  | Trial description |
|  | Select: data status: raw vs. processed (analysed) data |
|  | Institute |
|  | Country |
|  | Place name |
|  |  |
| 1. Population type & template design | 1. **Traits** |
| Taxonomy term | Selection of trait descriptors |
| Population type | When defining new trait descriptor, define: |
| Download .csv file template & fill out: | Trait name, Method, Scale |
| Species, Plant Line and/or Variety, Crop type, |  |
| Plant accession, Year produced, Originating Organisation |  |
|  |  |
| 1. Population Template submission | 1. **Scoring template design** |
| Submission of template | type of genetic material |
|  | Specify technical replicate numbers |
|  | Design factors (e.g. block, plot, rep etc.) |
|  |  |
| 1. Provenance | 1. **Scoring Template Submission** |
| Data owned by (optional) | Download .csv file template & fill out: |
| Data provenance (optional) | Plant scoring unit (= Sample_id), Plant accession, |
| Comments (optional) | Originating Organisation, Plant Line or Variety |
| Visibility - public/private | Submission of template |
|  |  |
|  | 1. **Trial Layout Submission** |
|  | Submission of trial layout image (optional) |
|  |  |
|  | 1. **Provenance** |
|  | See provenance fields in Population Submission |
|  |  |

Table S1 Overview of steps during the wizard-based Population and Trial (Trait Scoring) submission, naming all compulsory and some optional fields. All fields in the database can accessed and submitted to via the BIP-API, where the same fields are compulsory as for the wizard submission. The full list of database fields can be found in the API documentation (https://bip.earlham.ac.uk/api_documentation).
