# Supplementary material for: Introducing the Brassica Information Portal: Towards integrating genotypic and phenotypic Brassica crop data
Source: F1000Res. 2017 Nov 15;6:465. Originally published 2017 Apr 12. [Version 2] doi: 10.12688/f1000research.11301.2 (PMC5428495; doi:10.12688/f1000research.11301.2)
Supplement: Peer review history

# Peer review: Referee response for version 1

**DOI:** 10.5256/f1000research.12194.r22091

**Published:** 2017-05-09

**Authors:** Matthew M. Nelson

**Affiliations:** 1 Natural Capital and Plant Health, Royal Botanic Gardens Kew, Ardingly, UK

This manuscript describes a valuable resource for the Brassica research and breeding communities. It is logically and clearly written. 

 I felt that some of the methodology was inadequately cited. For example, on P4: the terms "PostgreSQL RDBMS technology”, “Ruby on Rails”, “Nginx” and “Elasticsearch” are used without any external references. Ideally scholarly or technical journal articles would be cited; if not available, then the publisher's name or project website.

 Some minor edits required:

 P3: The phrase ‘assist and help’ contains a tautology. Choose one of these terms, not both.

 P3: Replace ‘steming’ with ‘stemming’

 P6: Presumed typo in the header “0.1 Content navigation”?

I have read this submission. I believe that I have an appropriate level of expertise to confirm that it is of an acceptable scientific standard.

# Peer review: Referee response for version 1

**DOI:** 10.5256/f1000research.12194.r21824

**Published:** 2017-05-05

**Authors:** Cyril Pommier

**Affiliations:** 1 Research Unit in Genomics-Info (URGI) UR1164, French National Institute for Agricultural Research (INRA), Versailles, France

The rational and the objective of the Brassica Information Portal is well explained. Its complementarity with existing data repositories including genomic data repositories and tools and in particular its role as an important repository for Brassica Phenotyping Data is clear. Its relation to other Phenotyping Brassica repositories could be further discussed as perspectives.

 BIP makes good use of the current standards for plant phenotyping data, in particular MIAPPE and Cropontology. By being implicated in the current evolutions of MIAPPE, the BIP team demonstrate its will to ensure interoperability of this system with international standards. This is important for long term sustainability.

 The data submission process is well described. It shows that significant efforts have been made to ensure data quality and to ease data managers task. Data consistency between population, ontologies and trait datasets should be ensured with the chosen mechanisms. Data citation is well ensured by a DOI generation for datasets.

 BIP use modern and proven technological solutions, like Elasticsearch, Nginx web server, rich BIP Web Service API backing the whole user interface and allowing user or programatic interaction with the system.

 For the technical description of the software tool to be fully sound, the following points should be improved.

- The role of BIP in the BrAPI should be further described. In the Author Contributions, BrAPI is cited but it doesn't appears elsewhere in the text.
- Some technical description could be improved, for instance "universal search" isn't very clear.

This paper shows very well the development process of the BIP information system. The current status of the system is clear. Its perspectives could be further discussed regarding FAIR compatibility, Open Data including data licences policies (CC-by). Alongside this point, BIP team curent opinion on  distributed phenotyping information system, backed by the BrAPI, could be discussed.

I have read this submission. I believe that I have an appropriate level of expertise to confirm that it is of an acceptable scientific standard.

# Peer review: Referee response for version 1

**DOI:** 10.5256/f1000research.12194.r21825

**Published:** 2017-05-03

**Authors:** Christopher J. Rawlings

**Affiliations:** 1 Department of Computational and Analytical Sciences, Rothamsted Research, Harpenden, UK

The background to the development of the Brassica Information Portal and its origins in the CropStore system are well described. This paper is an update on the work in progress to deliver an integrated resource linking phenotype to genotype data for Brassica species.

 There are welcome new developments in the BIP including greater use of ontologies, in particular links with the MIAPPE initiative to capture phenotype in a systematic way and to adopt emerging metadata standards for crop phenotyping projects.

 A summary of the contents demonstrates that the BIP already comprises a significant scale repository for Brassica pre-breeding data and where relevant this is linked to SNP data visible with a genome browser.

 This paper is about the BIP as an information resource, rather than a publication of a new method. The paper provides a high level view of the technical implementation, reporting on adoption of the PostgreSQL relational database technology and the general use of Restful services as the basis for the data management operations in the BIP, including those that support improved interfaces for data loading and for browsing. A conceptual database schema is presented for the BIP database. 

 There are other areas of BIP that are adopting good practise in science data management, including the use of Orcid identifiers for user authentication and data search and retrieval using Elasticsearch technology.

 The paper provides overviews of key data management tasks and gives more information in the Supplementary material provided with the manuscript. There are also links to the Open Source repository Github where the software for BIP is available and to the example code for querying the data programmatically.

 Overall, this is an informative paper about the current state of the Brassica Information Portal. It is not a primary research paper, but rather a report of work in progress supporting the Brassica research community with a useful resource and software that could be re-used for other crops where there is a requirement for the management and dissemination of crop genotype-phenotype datasets.

 While there has been good progress so far towards developing the software so that the system supports open access to the software and data, it remains unclear to what extent the FAIR principles of data stewardship are being embedded in the BIP software. (e.g. Wilkinson et al ^1^). In particular, it would be helpful for the authors to describe their plans for exposing discoverability metadata and how they view the role of BIP as a “ FAIRport” for Brassica data.

I have read this submission. I believe that I have an appropriate level of expertise to confirm that it is of an acceptable scientific standard.

## References

[1] WilkinsonMDDumontierMAalbersbergIJAppletonGAxtonMBaakABlombergNBoitenJWda Silva SantosLBBournePEBouwmanJBrookesAJClarkTCrosasMDilloIDumonOEdmundsSEveloCTFinkersRGonzalez-BeltranAGrayAJGrothPGobleCGretheJSHeringaJ't HoenPAHooftRKuhnTKokRKokJLusherSJMartoneMEMonsAPackerALPerssonBRocca-SerraPRoosMvan SchaikRSansoneSASchultesESengstagTSlaterTStrawnGSwertzMAThompsonMvan der LeiJvan MulligenEVelteropJWaagmeesterAWittenburgPWolstencroftKZhaoJMonsB: The FAIR Guiding Principles for scientific data management and stewardship. *Sci Data*.2016;3: 10.1038/sdata.2016.18 160018 10.1038/sdata.2016.18 26978244PMC4792175
